# Developing predictive hybridization models for phosphorothioate oligonucleotides using high-resolution melting

**DOI:** 10.1371/journal.pone.0268575

**Published:** 2022-05-18

**Authors:** Siyuan S. Wang, Erhu Xiong, Sanchita Bhadra, Andrew D. Ellington

**Affiliations:** Department of Molecular Biosciences, Center for Systems and Synthetic Biology, College of Natural Sciences, The University of Texas at Austin, Austin, Texas, United States of America; Synthorx, UNITED STATES

## Abstract

The ability to predict nucleic acid hybridization energies has been greatly enabling for many applications, but predictive models require painstaking experimentation, which may limit expansion to non-natural nucleic acid analogues and chemistries. We have assessed the utility of dye-based, high-resolution melting (HRM) as an alternative to UV-Vis determinations of hyperchromicity in order to more quickly acquire parameters for duplex stability prediction. The HRM-derived model for phosphodiester (PO) DNA can make comparable predictions to previously established models. Using HRM, it proved possible to develop predictive models for DNA duplexes containing phosphorothioate (PS) linkages, and we found that hybridization stability could be predicted as a function of sequence and backbone composition for a variety of duplexes, including PS:PS, PS:PO, and partially modified backbones. Individual phosphorothioate modifications destabilize helices by around 0.12 kcal/mol on average. Finally, we applied these models to the design of a catalytic hairpin assembly circuit, an enzyme-free amplification method used for nucleic acid-based molecular detection. Changes in PS circuit behavior were consistent with model predictions, further supporting the addition of HRM modeling and parameters for PS oligonucleotides to the rational design of nucleic acid hybridization.

## Introduction

The programmability of nucleic acids for biotechnology and nanotechnology applications is based on the highly predictive thermodynamic properties of DNA and RNA hybridization, which can be well-approximated by the nearest-neighbor model [1–4]. In consequence, the stability of a given duplex can generally be accurately predicted from its sequence [5–11]. Typically, nearest-neighbor model parameters for nucleic acids are derived using UV-Vis spectrophotometry, relying on the hyperchromicity of single-stranded DNA and RNA to capture the transition from duplex to denatured strands. Melting temperatures and other thermodynamic values pertaining to the duplex are derived by fit to the melting profile. While such hyperchromicity methods can produce thermodynamic parameters that are broadly applicable to various predictions because they result from direct measurements of duplex melting, the material cost and low throughput of UV-Vis spectrophotometry can be prohibitive. This is particularly true in the case of expensive or precious non-canonical oligonucleotides. As a result, while nearest-neighbor parameters have been found for some non-canonical bases [12] and unnatural backbones [13, 14], many other broadly employed chemical modifications to DNA and RNA have yet to be similarly adapted to predictive models. The rational design of nucleic acid hybridization for both structure and function is therefore generally limited to the use of unmodified oligonucleotides.

High-resolution melting (HRM) represents a higher throughput and more cost-efficient method for quantifying duplex stability and consequently deriving predictive parameters. Many qPCR machines are also capable of collecting melting curve data and have software that can perform HRM analysis. In this method, sequence non-specific intercalating dyes such as EvaGreen or LC Green obviate the need for custom fluorescent probes or fluorophore-quencher modifications. Experiments can be scaled up in 96-well plates with volumes on the order of 10 microliters and as little as picomoles of material. HRM has been widely employed in molecular diagnostics to rapidly discriminate between near-identical sequences through shifts in melting temperatures, enabling applications such as single-nucleotide polymorphism genotyping and quantification of mosaicism [15, 16].

In this study, we assessed the feasibility of HRM as a method for determining the sequence-dependent thermodynamic parameters for phosphorothioate (PS) oligonucleotides. We designed sets of phosphodiester (PO) DNA oligonucleotide duplexes with sequences that maximally spanned the space of nearest-neighbor nucleotide pair parameters and determined the T_m_ of each duplex at various concentrations using HRM with EvaGreen intercalating dye. We fitted transition thermodynamic parameter enthalpy (ΔH), entropy (ΔS), and free energy (ΔG) to the collected T_m_ values using Van’t Hoff analysis and then derived approximate nearest-neighbor parameters using singular value decomposition. While a potential drawback to using HRM to characterize nucleic acid duplex thermodynamics is the introduction of systematic errors due to binding interactions with intercalating dyes, we find that it is possible to apply a linear correction to HRM-derived model predictions (i.e. ΔG_37_ = ΔG_37,HRM_—3.73 kcal/mol + 0.19 kcal/mol/base pair * sequence length) and thereby generate predictions comparable to those made by models derived from hyperchromicity data. Using HRM, predictive models for DNA duplexes containing PS modifications were fitted, PS modifications were incorporated into a DNA-based amplification circuit and changes to circuit behavior that corresponded to predictions were observed. HRM methods can therefore potentially accelerate the use of nucleic acid modifications in rationally designed oligonucleotides for a variety of applications, including for antisense oligonucleotide design.

## Materials and methods

### Reagents and oligonucleotides

All oligonucleotides were ordered from Integrated DNA Technology (IDT, Coralville, IA, USA). PS DNA oligonucleotides were produced through non-stereospecific chemical synthesis; as a result, PS oligonucleotides used in this study may contain either the R_p_ or S_p_ diastereomer at each modified position. All chemicals were purchased from Fisher Scientific (Waltham, MA, USA). Oligonucleotides used for model parameter determination are listed in **S1 Table in S1 File**, partially PS modified sequences are given in **S2 Table in S1 File**, and those used for catalytic hairpin assembly are listed in **S3 and S4 Tables in S1 File**. Oligonucleotides were stored at 100 μM in nuclease-free water at -20°C. Reactions were carried out in 1× NNE buffer (500 mM NaCl, 10 mM Na_2_HPO_4_, 1 mM EDTA, pH 7.0) for HRM experiments and 1× TNaK buffer (20 mM Tris-HCl, 140 mM NaCl, 5 mM KCl, pH 7.5) for catalytic hairpin assembly assays.

### Sequence design for parameter determination

Each sequence can be represented as a linear combination of nearest-neighbor nucleotide pairs [17]; the linear combinations of pairs that make up a set of sequences can be represented together as a stacking matrix. The duplex thermodynamic value (i.e. ΔG, ΔH, ΔS) of a given sequence is the sum of the contributions of each parameter in the duplex. Thus, in the example of ΔG, given a set of sequences represented by stacking matrix *A*, we can represent the duplex ΔG of all sequences in the set as a vector $\vec{b}$, where $\vec{b}$ is the product of the stacking matrix and the vector of all parameter ΔG contributions $\vec{x}$

$$\left[\begin{array}{ccc} n_{seq1,AA/TT} & n_{seq1,AT/TA} & \ldots \\ n_{seq2,AA/TT} & n_{seq2,AT/TA} & \ldots \\ \ldots & \ldots & \ldots \end{array}\right]\times \left[\begin{array}{c} \Delta G_{AA/TT}\\ \Delta G_{AT/TA}\\ \ldots \end{array}\right]=\left[\begin{array}{c} \Delta G_{seq1}\\ \Delta G_{seq2}\\ \ldots \end{array}\right]$$

The sequence set was designed to have a stacking matrix rank of 20 when considering a nearest-neighbor model for oligonucleotide duplexes of non-fixed length with 4 bases (i.e. 24 possible variables), which is the maximum rank attainable for this model [10, 17]. The final set contains 66 total sequences, including three 20-sequence subsets that independently attain rank 20 when considered under this model.

### T_m_ measurement, determination of thermodynamic values, and model fitting

Each duplex was annealed prior to melting experiments by adding equivalent amounts of top and bottom strands to obtain a final concentration of 25 μM and incubated for 5 minutes at 95°C followed by a 0.1°C/s ramp down to 20°C. The annealed sequences were used to prepare 4 replicate samples at various final concentrations (1, 2.5, 5, 7.5, 10, 15, 20 μM), and each sample was adjusted to contain 1× NNE buffer and 1× EvaGreen dye (20× EvaGreen dye in water purchased from Biotium, Hayward, CA). HRM data was collected in the Roche LightCycler96 qPCR machine (Roche Molecular Systems, Inc., CA, USA) at excitation 470 nm and emission 514 nm. The melting protocol was as follows: 5 minute incubation at 37°C, 0.1°C/s ramp up to 97°C with 10 readings/°C, and 2 minute incubation at 97°C. dF/dT (change in fluorescence signal over temperature) was calculated using the Roche LightCycler Software version 1.1.0 (Roche Diagnostics International) by selecting “Add Analysis” and “T_m_ calling”. T_m_ is defined as the peak of the–dF/dT curve, and samples without distinct peaks were excluded from the analysis. We used linear regression to fit the melting data to the equation

$$\frac{1}{T_{m}}=\frac{R}{\Delta H}ln\left(\frac{C_{T}}{4}\right)+\frac{\Delta S}{\Delta H}$$

to estimate duplex ΔH, ΔS, and by extension, ΔG. ΔG was extrapolated to 50°C to minimize heat capacity changes of unfolding. Values of ΔS or ΔG were adjusted to 1 M NaCl during the fit using the salt correction reported in [5]. Unadjusted values are reported in the Supplemental Data. A total of 4 sequences in the PO-PO dataset, 1 in the PS-PO dataset, and 2 in the PS-PS showed high ΔH error (>30% of fitted ΔH value) were removed on the basis that high error during Van’t Hoff analysis suggests either non-two-state behavior or incorrect concentration. In each dataset, the set of remaining sequences maintained the same rank for stacking matrices determined as previously described. All errors reported are standard deviations of the parameter fits. Sequence ΔS and ΔH variances for each sequence were determined by regression and used to calculate ΔG variances as described in [18].

For each model, the sequence variances were transformed into the parameter basis, resulting in a covariance matrix (*C*_*NN*_). To allow us to drop covariances between parameters while not underestimating the error, we found the smallest diagonal covariance matrix *C’*_*NN*_ in the parameter space such that the matrix inequality *C*_*NN*_ ≤ *C’*_*NN*_ holds. Variances derived from *C′*_*NN*_ are guaranteed to be equal to or overestimate the error on parameters; we report the standard deviations of these parameters. We performed all data analyses using Python, including linear regression to the Van’t Hoff equation (*scipy*.*optimize*.*curve_fit*), singular value decomposition (*numpy*.*linalg*.*svd*), minimization of residual sum of squares (*scipy*.*minimize*), and convex optimization for finding *C’*_*NN*_ (*cvxpy*).

### Catalytic hairpin assembly fluorescence kinetic reading

A 2.5 μM stock of reporter complex was prepared by mixing 2.5 μL of RepF (100 μM stock in 1× TNaK buffer), 5 μL of RepQ (100 μM stock in 1× TNaK buffer), 10 μL of 10× TNaK buffer, and dH_2_O to reach a final volume of 100 μL, followed by annealing. A two-fold excess of RepQ was added to ensure efficient quenching of RepF, which is not expected to interfere with readout. Prior to the experiments, folded solutions of hairpin 1 (H1) at 5 μM (5 μL of 100 μM stock solution, 10 μL of 10× TNaK buffer, and 85 μL of dH_2_O) and hairpin 2 (H2) at 10 μM (10 μL of 100 μM stock solution, 10 μL of 10× TNaK buffer, and 80 μL of dH_2_O) were individually prepared from their respective 100 μM stock solutions by a 5 minute incubation at 95°C followed by a 0.1°C/s ramp down to 20°C. Reaction mixtures (total volume of 25 μL) contained the following final concentrations in 1× TNaK buffer: 200 nM folded H1, 400 nM folded H2, 50 nM annealed reporter complex, 1 μM polyT (dT_21_), and various concentrations of the catalyst strand (500 nM, 250 nM, 125 nM, and 50 nM). Reaction mixtures were loaded to a 96-well plate and immediately transferred to the LightCycler96 plate reader (Roche Molecular Systems, Inc., CA, USA) for fluorescence measurements conducted at 37°C or higher (excitation: 470 nm, emission: 514 nm).

## Results

### Derivation of thermodynamic parameters with high-resolution melting

To derive approximate thermodynamic parameters using HRM, we designed a set of sequences that achieved the highest number of linearly independent sequences possible given constraints between parameters. In order to design the most broadly applicable sequence sets, we first considered a nearest-neighbor model for oligonucleotide duplexes of non-fixed length and 4 possible bases that makes no assumptions about symmetry and includes parameters for terminal ends (**S1 Fig in S1 File**). This model includes a total of 24 parameters and has a highest attainable stacking matrix rank of 20 [10]. To evenly represent all parameters in sequence space, we designed 3 sets of sequences that each attained this rank and combined these sets to produce a total of 66 sequences. The sequences ranged between 12 and 30 bases in length, with predicted T_m_ values between 50°C and 80°C, as this suited the temperature range of the qPCR machine used for analysis (37°C to 98°C). Sequences were also designed to have secondary structures that were less stable than -1 kcal/mol at 37°C and 0.5 M NaCl, as calculated by NUPACK [19].

**Fig 1:**
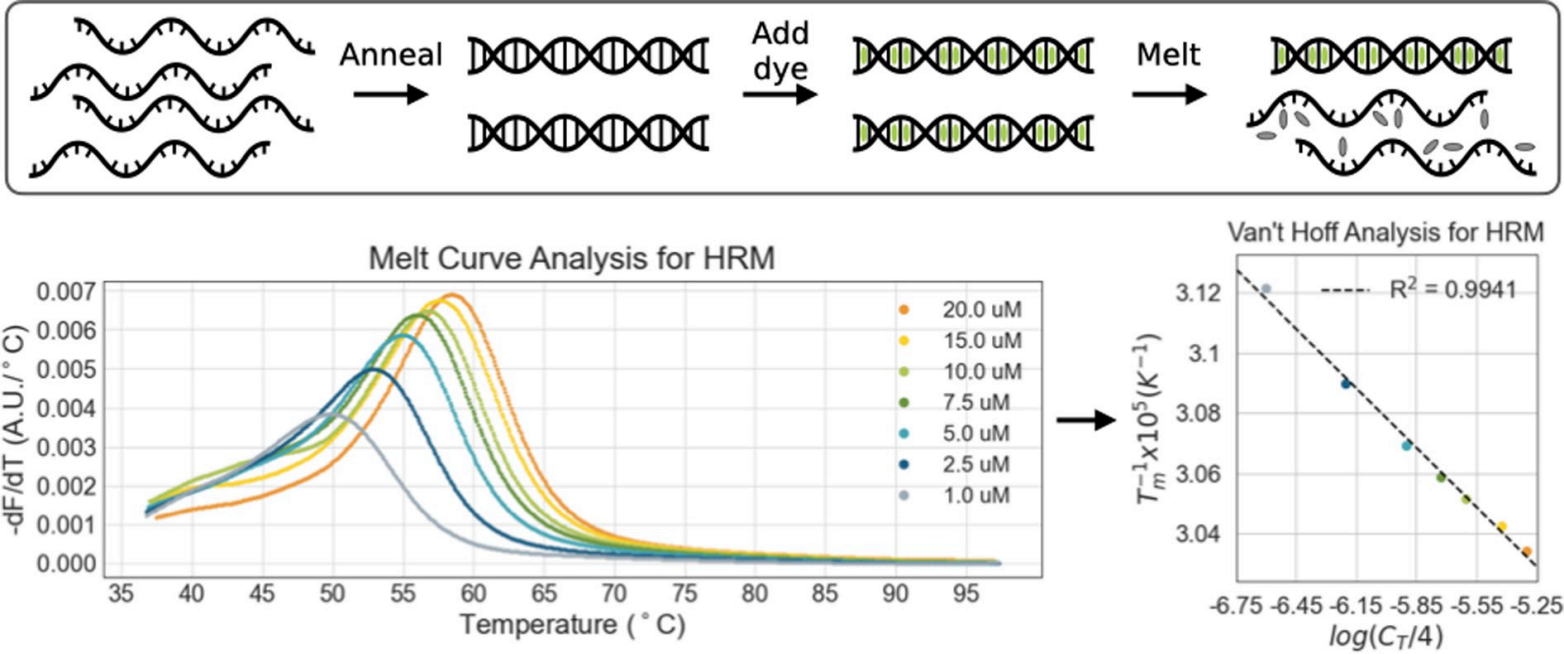
High-Resolution Melting (HRM) pipeline for determining duplex stability. Peak change in fluorescence (dF/dT) indicates melting temperature. Thermodynamic parameters are derived from Van’t Hoff analysis on HRM data. Since all sequences are non-self-complementary, 1/T_m_ is plotted against ln(C_T_/4).

We performed HRM with an EvaGreen intercalating dye on thermally annealed duplexes that comprised each sequence in the designed set and its complement, at concentrations ranging from 1 μM to 20 μM. For each concentration, the T_m_ was determined as the peak in the -dF/dT of the melting curve. We applied linear regression to the T_m_ series using the Van’t Hoff equation and thereby determined ΔH, ΔS, and ΔG_50_ values (after adjusting to 1 M NaCl as reported by [5]) (**Fig 1**). In general, R^2^ values were greater than 0.95. Experimentally derived, non-salt-adjusted ΔH, ΔS, and ΔG_50_ values are reported in the **S1 File**.

To determine individual nucleotide pair parameters for ΔH, ΔS, and ΔG_50,_ we used singular value decomposition with the experimentally derived thermodynamic values. Because the choice of included parameters can affect the quality of predictions, we assessed different models for accuracy. For duplexes that consist of chemically identical backbones (i.e. that are “symmetrical” about the base pairing axis, such as native DNA; PO-PO) there are a total of 10 internal nucleotide pairs that remain unique when inverted (e.g. 5’CA/3’GT versus 5’TG/3’AC; **S1 Fig in S1 File**). Additionally, each duplex contained one of 4 possible terminal nucleotides at either end. We therefore considered whether including dedicated terminal nucleotide parameters (represented by fictitious E and E’ nucleotides in the model) might improve predictions. We performed leave-one-out cross-validation by systematically leaving out each sequence and predicting its ΔG and T_m_ using thermodynamic parameters fitted to the remaining n-1 sequences with and without terminal parameters. We found that the two models performed similarly when predicting ΔG (out-sample root mean square error of 0.84 kcal/mol without and 0.86 kcal/mol with terminal parameters), but inclusion of terminal parameters significantly improved T_m_ prediction, particularly for shorter or longer sequences (1.88°C versus 1.17°C; **S2 Fig in S1 File**). These results suggest that terminal parameters can account for global effects not captured by internal nucleotide parameters and improve predictions in our model.

Parameters for ΔH, ΔS, and ΔG_50_ for fitted PO-PO internal nucleotide pairs and terminal nucleotides are shown in **Table 1**; non-salt-adjusted parameters derived from experimental conditions are reported in **S5 Table in S1 File**. Since duplexes were predicted to have melting temperatures within a 50–80°C range, the reported ΔG was extrapolated to 50°C (ΔG_50_) to minimize the impact of heat capacity changes on unfolding. Although errors (standard deviation) for fitted ΔH and ΔS parameters were high, the fact that ΔH and ΔS are highly correlated led to much smaller errors for the derived ΔG parameters (which rely on entropy-enthalpy compensation) [18]. The rank of the stacking matrix changes based on the choice of model for the same set of sequences—for instance, stacking matrices for the asymmetrical-terminal and the symmetrical-terminal models have rank 20 (maximum 24) and 12 (maximum 14), respectively. Due to sequence restraints, the stacking matrices cannot have the maximum rank (i.e. are underdetermined); in other words, sets of fitted parameters for these models are non-unique solutions [10]. Thus, each set of fitted parameters is one among infinite possible solutions, and any such solution would give the same prediction for any sequence [5].

**Table 1:** Approximate thermodynamic parameters derived from HRM data.

Nucleotide Pairs (PO-PO)	ΔG_50_ (kcal/mol)	ΔH (kcal/mol)	ΔS (cal/K/mol)
AA/TT	-0.83±0.14	-8.10±1.68	-22.5±4.8
AT/TA	-0.56±0.10	-5.53±1.35	-15.4±3.9
TA/AT	-0.58±0.12	-6.40±1.49	-18.0±4.3
CA/GT	-0.95±0.15	-6.89±1.70	-18.4±4.8
GT/CA	-0.94±0.15	-7.12±1.88	-19.1±5.3
CT/GA	-0.94±0.14	-7.51±1.63	-20.3±4.6
GA/CT	-0.88±0.14	-6.51±1.84	-17.4±5.3
CG/GC	-1.62±0.16	-10.81±2.03	-28.5±5.8
GC/CG	-1.76±0.16	-12.68±1.98	-33.8±5.6
GG/CC	-1.09±0.15	-6.09±1.71	-15.5±4.8
EA/ET	0.49±0.40	20.73±4.98	62.6±14.2
AE/TE	0.48±0.40	20.20±4.89	61.0±13.9
EC/EG	0.63±0.40	21.07±4.93	63.2±14.0
CE/GE	0.43±0.40	18.09±4.84	54.7±13.8

All reported values are adjusted to 1 M NaCl and 50°C. PO-PO = Phosphodiester-phosphodiester duplexes. Errors are defined as the standard deviations of the parameter fits. Parameter values are non-unique solutions from the model fit.

**Fig 2:**
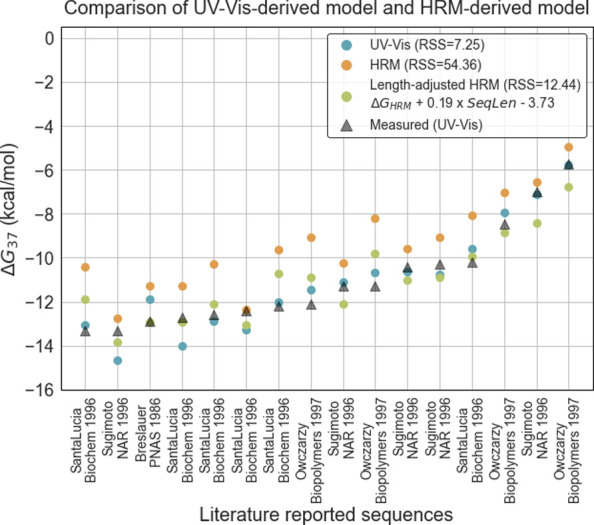
Comparison of ΔG predictions made by the HRM-derived model and reported UV-Vis models for 16 literature reported sequences. Sequences are shown in **S6 Table in S1 File**. RSS = residual sum of squares.

Parameter values from the HRM-derived model were on average higher (i.e. less stabilizing) than values reported in previous nearest-neighbor models. The increase in T_m_ is larger with lower dye/base pair ratio (**S3 Fig in S1 File**), as has been previously observed [20]. Differential intercalation at different DNA concentrations or lengths shifts the slope of the fitted curve, resulting in a higher (i.e., less stable) observed ΔG. We empirically corrected these effects in part by applying a length-dependent correction to the predictions made by the HRM model. We selected a total of 16 duplexes whose ΔG values had been previously determined from hyperchromicity measurements, ranging from 10 to 16 nucleotides in length (**S6 Table in S1 File**) [6, 9, 21, 22]. We avoided sequences containing homopolymer runs greater than 4 bases, as our own sequence designs originally excluded these. We used ΔH and ΔS parameters from our HRM model to predict ΔG_37_ of each sequence (ΔG_37_-HRM) and fitted a linear length-dependent correction that adjusted this value to match as closely as possible reported values extracted from melting as assessed via hyperchromicity. An equation to correct for dye intercalation, ΔG_HRM_ + A * SequenceLength + B, was fitted to minimize the residual sum of squares (RSS) value between predicted and reported values, resulting in values 0.19 and -3.73 kcal/mol for A and B, respectively. The corrected model had a RSS of 12.44 compared to 7.25 for previously established hyperchromicity models [5], a great improvement over the uncorrected model, which had a RSS of 54.36 (**Fig 2**). These corrections apply to models determined using a concentration of 1X EvaGreen (1.25 μM), and models determined at other dye concentrations would result in quantitatively different shifts, although relative stability predictions would remain similar. In fact, to investigate how the derived ΔG is impacted by dye concentration, we determined ΔG for two sequences at dye concentrations 0.5X, 1X, and 2X. We found that the measured ΔG was closest to the hyperchromicity model predictions at a 2X dye concentration (**S4 Fig in S1 File**).

### Predictive models for duplexes with fully phosphorothioate-modified oligonucleotides

Having proved the basic method’s applicability, we attempted to establish approximate models for duplex stability with DNA strands containing entirely phosphorothioate (PS) linkages, for which sequence-dependent parameters had not been previously determined. We anticipated that PS duplexes should be well-approximated by nearest-neighbor models since the phosphorothioate modification does not alter the structure of nucleobases and base-stacking has been shown to be the major energetic contributor to helix stability [1, 2].

We studied two types of duplexes: a PS DNA strand paired with an opposing PS DNA strand (PS-PS), and a PS DNA strand paired with a PO DNA strand (PS-PO). Our sequence sets included the same 66 sequences described previously for PO-PO. Because PS-PO duplexes are hybrid duplexes that are not “symmetrical” about the base pairing axis (unlike PO-PO and PS-PS), a larger set of parameters was needed, since no nucleotide pair was redundant. This “asymmetrical” model contained a total of 16 internal nucleotide pair parameters and 8 terminal nucleotide parameters (**S1 Fig in S1 File**). We again performed leave-one-out cross-validation to compare the fit of the symmetrical model with and without terminal parameters for PS-PS duplexes (**S4 Fig in S1 File**), and the asymmetrical model with and without terminal parameters for PS-PO duplexes (**S5 Fig in S1 File**). As was previously observed for PO-PO duplexes, the inclusion of the terminal parameters significantly improved T_m_ prediction accuracy (RMSE of 3.04°C to 1.60°C for PS-PS and 2.84°C to 1.73°C for PS-PO). Addition of terminal parameters largely improved ΔG prediction in PS-PS (RMSE of 0.69 kcal/mol to 0.38 kcal/mol), but not in PS-PO (0.63 kcal/mol to 0.62 kcal/mol). Fitted parameters for the symmetrical model for PS-PS and for the asymmetrical model for PS-PO duplexes are shown in **Tables 2** and **3**, respectively; non-salt-adjusted parameters derived from experimental conditions (1× NNE buffer) are reported in **S7 Table** and **S8 Table in S1 File**, respectively. Terminal parameters are clearly important inclusions to HRM-derived duplex stability models for accurate prediction of thermodynamic properties such as ΔG and T_m_. Interestingly, we observed an increase in stability of the terminal parameters as the duplex included more fully PS strands: an average of 0.51, -0.52, and -1.25 kcal/mol for each terminal nucleotide for PO-PO, PS-PO, and PS-PS, respectively. This suggests that the fitted internal parameters slightly overestimated the destabilization due to modification and required a global correction in the form of terminal parameters.

**Table 2:** Approximate thermodynamic parameters for PS-PS (phosphorothioate-phosphorothioate) duplexes derived from HRM data.

Nucleotide Pairs (PS-PS)	ΔG_50_ (kcal/mol)	ΔH (kcal/mol)	ΔS (cal/K/mol)
AA/TT	-0.26±0.03	-4.36±0.77	-12.7±2.3
AT/TA	-0.16±0.02	-3.64±0.52	-10.8±1.5
TA/AT	-0.11±0.01	-1.93±0.53	-5.6±1.6
CA/GT	-0.52±0.02	-5.52±0.57	-15.5±1.7
GT/CA	-0.50±0.03	-3.95±0.75	-10.7±2.3
CT/GA	-0.50±0.03	-4.16±0.64	-11.3±1.9
GA/CT	-0.57±0.03	-5.07±0.90	-13.9±2.7
CG/GC	-1.06±0.04	-6.16±1.01	-15.8±3.0
GC/CG	-1.04±0.04	-6.90±0.77	-18.1±2.3
GG/CC	-0.85±0.03	-5.09±0.83	-13.1±2.5
EA/ET	-1.30±0.08	3.24±2.05	14.0±6.2
AE/TE	-1.28±0.08	4.18±2.02	16.9±6.1
EC/EG	-1.20±0.08	1.47±1.98	8.3±5.9
CE/GE	-1.20±0.08	0.64±2.05	5.7±6.1

All reported values are adjusted to 1 M NaCl and 50°C. All internal nucleotide parameters have PS linkages both in the top nucleotide pair and in the bottom pair (e.g. 5’A*A/3’T*T). Errors are defined as the standard deviations of the parameter fits. Parameter values are non-unique solutions from the model fit.

**Table 3:** Approximate thermodynamic parameters for PS-PO (phosphorothioate-phosphodiester) duplexes derived from HRM data.

Nucleotide Pairs (PS-PO)	ΔG_50_ (kcal/mol)	ΔH (kcal/mol)	ΔS (cal/K/mol)
AA/TT	-0.52±0.20	-5.81±3.12	-16.4±9.1
AT/TA	-0.42±0.10	-5.64±1.69	-16.2±4.9
AC/TG	-0.88±0.11	-8.66±1.85	-24.1±5.4
AG/TC	-0.71±0.09	-6.13±0.85	-16.8±2.3
TA/AT	-0.30±0.10	-3.86±1.90	-11.0±5.6
TT/AA	-0.49±0.07	-5.87±0.64	-16.7±1.7
TC/AG	-0.64±0.16	-6.13±2.71	-17.0±7.9
TG/AC	-0.77±0.15	-7.28±2.75	-20.2±8.0
CA/GT	-0.82±0.17	-7.23±2.67	-19.8±7.7
CT/GA	-0.66±0.19	-6.30±3.21	-17.5±9.4
CC/GG	-0.91±0.11	-5.57±1.57	-14.4±4.5
CG/GC	-1.21±0.18	-8.07±3.09	-21.2±9.0
GA/CT	-0.85±0.12	-7.92±2.38	-21.9±7.0
GT/CA	-0.61±0.14	-5.46±2.27	-15.0±6.6
GC/CG	-1.15±0.15	-6.63±2.26	-17.0±6.6
GG/CC	-1.09±0.17	-7.75±2.81	-20.6±8.2
EA/ET	-0.57±0.40	13.64±6.63	44.0±19.3
AE/TE	-0.54±0.37	15.07±6.01	48.3±17.5
ET/EA	-0.59±0.39	14.87±6.29	47.8±18.3
TE/AE	-0.56±0.38	14.72±6.18	47.3±18.0
EC/EG	-0.58±0.33	10.31±5.35	33.7±15.6
CE/GE	-0.55±0.38	10.49±6.30	34.2±18.4

All reported values are adjusted to 1 M NaCl and 50°C. All internal nucleotide parameters have a PS linkage between the top nucleotide pair (e.g. 5’A*A/3’TT). Errors are defined as the standard deviations of the parameter fits. Parameter values are non-unique solutions from the model fit.

It should be noted that our study used non-stereospecific PS oligonucleotides, yielding a chemical mix of 2^n^ different strands for sequences of length *n*. The large number of different compositions (but not sequences) is expected to produce a broader melting transition than PO-PO or stereospecific PS-PS melting, because presumably each composition has a slightly different melting temperature [23]. However, the impact of R_p_ and S_p_-stereoisomers on duplex stability is largely sequence-dependent, with pyrimidine-rich sequences having greater discrepancies between the two stereoisomers than balanced sequences [23, 24]. As the purine-pyrimidine compositions of our sequences were balanced, we did not expect a large effect, and indeed we observed melting derivative peaks for PS-PS and PS-PO melting that were only slightly broader than for PO-PO (data not shown).

### Predictive models for duplexes with partially phosphorothioate-modified oligonucleotides

Next, we investigated how to best model the thermodynamics of duplexes containing strands that contain a mix of PO and PS linkages. To increase the generality of our methods, we selected 2 new sequences unrelated to the previous 66 we had used and designed a set of partially PS-modified strands for each sequence ranging from 1 to 9 modifications (**S2 Table in S1 File**). We combined these partial-PS strands with either fully-PO or fully-PS complement strands to produce 10 duplexes that varied in the number of total PS modifications: from 0 (i.e. two fully PO strands); to 1, 4, 9, and 19 (i.e., a fully PO top strand with a fully PS bottom strand and vice versa); and ultimately to 20, 23, 28, 38 (i.e., two fully PS strands). The ΔG values of each partially-modified duplex were once again experimentally determined using HRM at a range of concentrations. To predict the ΔG of duplexes with partially-modified strands, we used the parameters from models fitted without terminal parameters, since individual modifications likely have unique impacts on global stability, and our terminal parameter models were based on fits for the global stability of duplexes containing fully phosphorothioate-modified strands.

**Fig 3:**
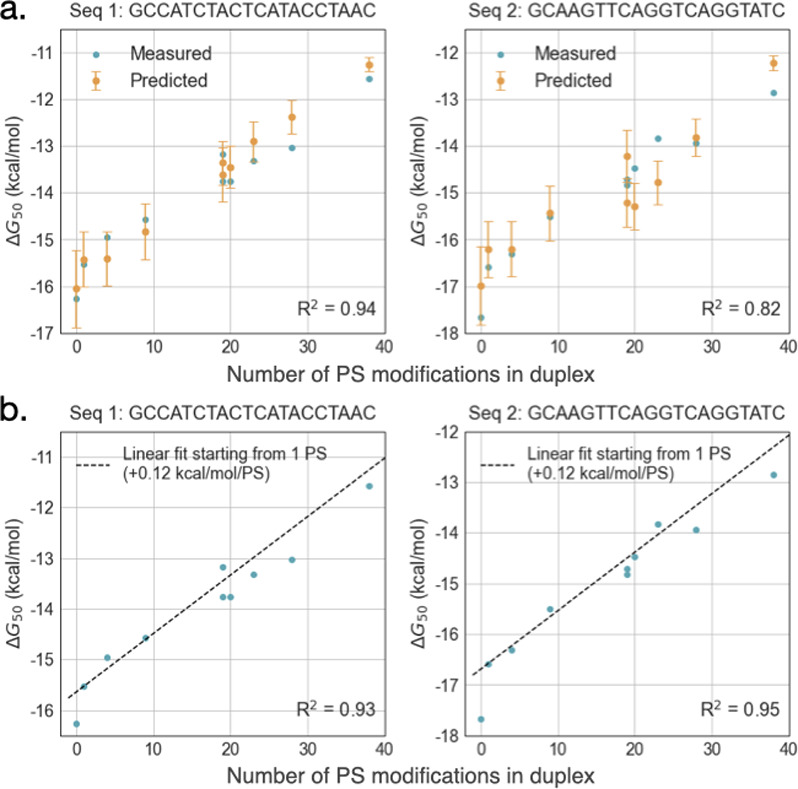
Predicting duplex stability of partially PS-modified duplexes as a function of sequence (a) or independently of sequence (b). (a) Duplexes with partially-modified strands were considered as a linear combination of PO-PO (symmetrical), PS-PO (asymmetrical), and/or PS-PS (symmetrical) nucleotide pairs and ΔG parameters of these pairs across the three backbone conditions were used to predict the overall duplex stability. Errors are calculated using the variances of the parameter estimates. (b) Data from fully PS-PO or PS-PS duplexes was used to determine the average energetic contribution of a single PS backbone (0.12 kcal/mol/PS). PO-PO duplexes (points at x = 0) are not included in the R^2^ shown for sequence-independent predictions due to the large gap in ΔG_50_ between x = 0 and x = 1 seen in both sequences tested. Positions of PS linkages in the included duplexes are shown in **S2 Table in S1 File**.

Comparing the measured and predicted ΔG values, we found that the model predicted the stability of the partially-PS duplex fairly well, resulting in an R^2^ of 0.94 and 0.82 for the two sequences tested (**Fig 3A**). Predictions were more accurate for duplexes in which fewer than half of all linkages were PS. Across all sequences in our set, we found that PS linkages resulted in an energetic difference of 0.12 ± 0.04 kcal/mol per modification, on average (**Fig 3B**).

The stabilities of partially-modified duplexes can thus be approximated in a sequence-dependent manner by nearest-neighbor type models, with a few caveats. First, transitions from one phosphate backbone to the other may result in energetic penalties that depend on a sequence context beyond nearest neighbors, since more modifications will result in an overall change in structure. This was best seen by the departure in prediction accuracy with increasing phosphorothioate modifications. Second, the lack of terminal parameters means that predictions will only hold true for a limited range of sequences. In the absence of terminal parameters specifically determined for duplexes at various levels of modification, using internal parameters alone to make predictions will cause shorter partially-modified duplexes to proportionally depart greater from experimental values.

### Predicting the impact of phosphorothioate modification on rationally designed nucleic acid circuits

Rationally designed nucleic acid systems have been used for a variety of applications, including enabling sensitive detection of analytes, precise assembly of nanoscale structures, and even chemical computation [25–27]. This programmability comes in part from the fact that experimental nucleic acid hybridization parameters often closely match theory, allowing accurate designs.

As an example, catalytic hairpin assembly (CHA) is an *in vitro* DNA-based signal amplification reaction capable of achieving up to hundreds-fold amplification of nucleic acid inputs [28, 29], making it potentially useful for diagnostic applications [30]. CHA designs to date have derived in large measure from predictions by programs such as NUPACK [19] that in turn rely on experimentally determined nearest-neighbor parameters. By modifying the sequence of key regions, CHA circuits have been engineered to operate at various temperatures [31] and to have reduced background leakage [32, 33].

While changes to circuit stability can be achieved by introducing mismatches or shortening sequence domains, modification of the backbone with phosphorothioates could also serve to destabilize hybridization of a given duplex relative to a fully phosphodiester counterpart. For example, the use of PS modifications (combined with additives such as single-stranded DNA-binding proteins and urea) has already enabled enzyme-mediated isothermal amplifications to operate with high specificity at lower temperatures [34]. Moreover, PS modifications should also prove useful for imparting nuclease resistance to DNA circuits mixed with biological samples [35].

**Fig 4:**
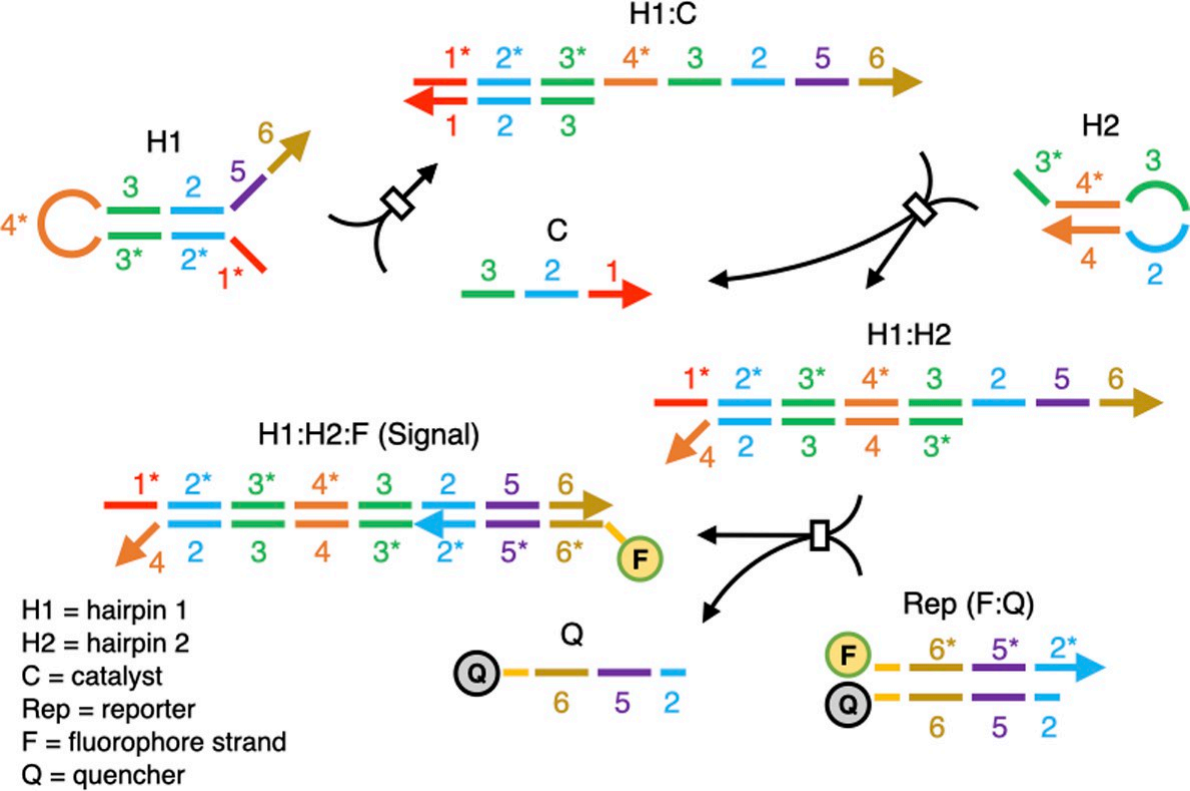
Reaction diagram of catalytic hairpin assembly. In this diagram, domains (short sequence segments) are indicated by number. Asterisks indicate sequence complements. Complexes of multiple strands are denoted with a colon. Key assemblies of strands are shown, while intermediate complexes involved in strand exchange and displacement reactions (e.g. domain 1 binding to domain 1* between C:H1, invasion of domains 2 and 3 on C into the H1 stem region, and unfolding of H1 by dissociation between the domains comprising the stem) are omitted (represented as boxes). Single-stranded catalyst (C) binds to Hairpin 1 (H1) via the single-stranded domain 1, and can initiate strand exchange and displacement with domains 2 and 3. The newly exposed single-stranded domain 3 then binds to the partially single-stranded domain 3* on Hairpin 2 (H2), again resulting in strand exchange and displacement, ultimately yielding H1:H2 (which is more stable than H1:C). The displaced C strand becomes available to catalyze another reaction. The reaction can be followed via a hemi-duplex reporter (Rep) where strand exchange and displacement of a fluorescent oligonucleotide conjugate from a quenching oligonucleotide conjugate is initiated by binding of domain 2 in the H1:H2 product.

**Fig 5:**
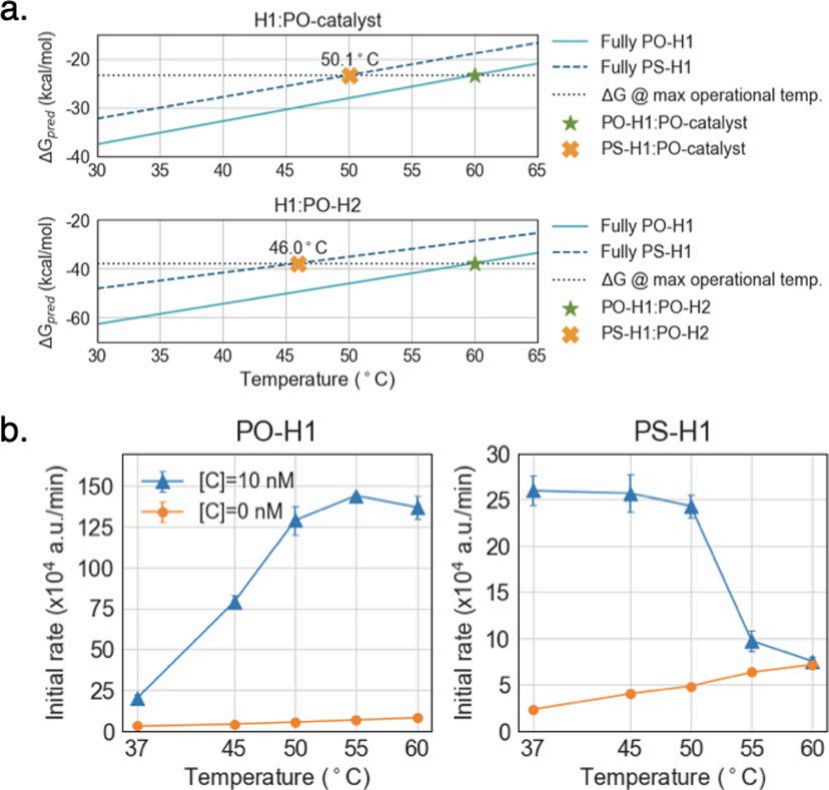
HT-CHA with PO- and PS-H1. H1 strand backbones are either fully PO or PS. (a) Duplex stability predictions for interactions involving PO-H1 or PS-H1 (hybridized region only, symmetrical model). Gray dotted line indicates the target stability or the duplex stability of PO-H1:catalyst or PO-H1:H2 at 60°C, the temperature for which the HT hairpins were originally designed [31]. (b) Initial rates of HT-CHA with PO-H1 or PS-H1 at various incubation temperatures. Catalyst strand is fully PO.

To further investigate whether and how PS modifications can impact circuit design, we generated a catalytic hairpin assembly (CHA) circuit that contained a hairpin (H1) that was fully phosphorothioate-modified (PS-H1) (**Fig 4** and **S3 Table in S1 File**). This circuit was based on a previously published high-temperature CHA (HT-CHA) circuit with an operating temperature of 60°C [31]. We predicted that the circuit would now have a lower effective temperature range, and that its performance could be predicted via the models we have developed. In greater detail, at the maximum operating temperature of 60°C, the unmodified intermediate (i.e. PO-H1:catalyst complex) and product (i.e. PO-H1:PO-H2 complex) species exhibit duplex stabilities of -23.3 kcal/mol and -37.8 kcal/mol in the hybridized region, respectively. Our model predicted that the modified versions of these complexes would have these same stabilities at 50.1°C (PS-H1:catalyst) and 46.0°C (PS-H1:PO-H2) (**Fig 5A**), suggesting that the circuit with PS-H1 would have a maximum operating temperature of around 50°C. In fact, when CHA was carried out with PS-H1 a decrease of activity beyond 50°C was observed (**Fig 5B**), in accord with modeling. A much lower overall signal was also observed with PS-H1 than with PO-H1 (e.g. peak activity of 25 a.u./min compared to 150 a.u./min). This was likely due to the reduced stability of the H1:Reporter complex as a result of phosphorothioate modifications to the H1 strand.

**Fig 6:**
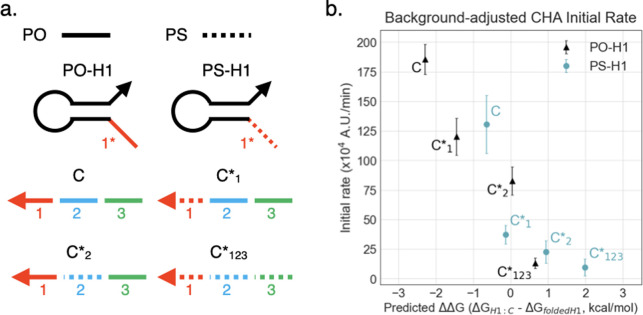
Introducing PS backbones to select domains in LT-CHA. (a) Modified parts used in LT-CHA circuit. (b) Observed initial rate of LT-CHA plotted against predicted change in free energy using modified-domain 1 hairpins with various modified versions of the catalyst strand. PO-H1 = hairpin 1 with only phosphodiester backbones. PS-H1 = hairpin 1 with PS modifications on domain 1*. C*_n_ = catalyst strand with PS on domain(s) n.

We then tested how smaller-scale PS modifications, such as modification of individual domains, can impact circuit behavior. To this end, we started with a previously developed low-temperature CHA (LT-CHA) circuit designed for operation at 37°C [31] as a starting point and generated versions of LT-CHA circuits with strands that contained one or more PS-modified domains. We chose to modify LT-CHA since LT-CHA components are less stable and therefore more sensitive than their high-temperature counterparts to small energetic penalties (i.e., ~0.12 kcal/mol per PS modification). These include a catalyst strand with a PS domain 1 (C*_1_), a catalyst strand with a PS domain 2 (C*_2_), a fully-modified catalyst strand (C*_123_), and a hairpin 1 with a PS toehold (PS-H1*_1_), as well as their PO counterparts (**S4 Table in S1 File** and **Fig 6A**). In the first step of CHA, the toehold of H1 binds to the single-stranded catalyst, and H1 is unfolded by the catalyst to form the H1:catalyst complex. Thus, the H1:catalyst complex must be energetically favored over the folded H1 structure to drive the reaction forward. To show the predictive power of our model, we estimated the difference in duplex stability between the hairpin:PS catalyst complexes and the folded PO hairpin (i.e. ΔΔG = ΔG-H1:C– ΔG_Folded_-PO-H1), which should correlate with circuit activity. In accord with PS destabilization and our model, a loss of activity was expected for CHA with PS-modified components.

In fact, the initial activity rates of chemically modified CHA circuits showed a good correlation with respect to ΔΔG (**Fig 6B**). For example, modifying domain 1 in only hairpin 1 of CHA with PS residues increased ΔΔG of the PS-H1:C complex to -0.65 kcal/mol and resulted in 75% of the original CHA activity. In contrast, modifying domain 1 in both hairpin 1 and the catalyst strand increased ΔΔG to -0.13 kcal/mol (i.e. only slightly favoring the forward reaction) and showed 20% of the original activity.

## Discussion

In this work, we carried out HRM experiments to develop approximate thermodynamic models for PO-PO, PS-PO, and PS-PS DNA duplexes, the latter two of which do not yet have published sequence-dependent models. Based on our analysis, T_m_ determination by HRM with the EvaGreen intercalating dye resulted in models that slightly underestimated the stability of duplexes (ie. predicted higher ΔG values). While part of the skew may be due to concentration-dependent dye intercalation, simply assuming a linear relationship between possible dye-binding positions (correlating with the total number of base-pairs) and the degree of destabilization allowed adjustments to be made, to the point where predictions were similar to those derived from UV-Vis hyperchromicity models. For models derived at 1X dye concentration, a correction of 0.19 kcal/mol/base pair sufficiently corrects ΔG_37_ predictions. A different factor would append at other dye concentrations (**S4 Fig in S1 File**), but the relative stabilities of duplexes with different sequences would still be predicted correctly.

More generally, there are notable differences between HRM and UV-Vis measurements that should be taken into account when fitting model parameters. The indirect nature of HRM allows high-throughput T_m_ measurements (i.e., compatible with 96-well plates) and relatively low concentrations (down to 1 μM oligonucleotide), resulting in more rapid and scalable model development. However, T_m_-HRM (the T_m_ defined by HRM) must be derived from the -dF/dT plot rather than by regression curve fitting or baseline extrapolation methods [36–39] typically used to determine T_m-_UV-Vis (the T_m_ defined by UV-Vis; the value at which half of all duplexes are bound). This is because curve fitting and baseline extrapolation are not sensitive enough to detect the duplex-to-single-stranded transition in HRM data at lower concentrations. Overall, this results in a ~1–2°C difference between T_m_-HRM and T_m_-UV-Vis [36]. HRM-based models therefore trade off opportunities for rapid and high-throughput modeling with lower accuracy. Depending on ultimate applications, T_m_−HRM should prove useful for quickly generating models for the increasing range of chemistries available to oligonucleotides. This would be especially helpful for characterizing backbone or sugar ring modifications that introduce a new degree of freedom that, in conjunction with nucleobase sequence, might require a combinatorially large (and synthetically intractable) set of duplexes to fully characterize.

By demonstrating that phosphorothioate duplexes, like phosphodiester duplexes, can be represented by a nearest-neighbor type model, we set the stage for the development of predictive models that can inform the designs of modified sequences that contribute to practical applications, such as nucleic acid circuitry. Our results showed that duplex stability decreases with an increasing number of modifications, with each modification resulting in an average energetic penalty of 0.12 kcal/mol. Destabilization via phosphorothioate modification was shown to affect circuit dynamics in a predictable manner and therefore provides a design strategy beyond merely editing sequence. In addition, considering that PS modifications have been regularly used in the design of therapeutic antisense oligonucleotides [40], our predictive models may narrow the range of designs, thereby reducing time and cost for testing candidates. For example, ATL1102 is a 20-nts antisense oligonucleotide designed for treatment of multiple sclerosis that is fully phosphorothioate-modified and additionally includes 2’-O-(2-methoxyethyl) modifications and methylated cytosine and uracil bases [41, 42]. Based on the PS-PO HRM model and assuming a physiological sodium concentration of 141 mM [43] and an oligonucleotide concentration of 10 nM, for a PS-PO duplex of the same sequence with no additional modifications we predict a T_m_ of 37.0°C, which is physiological temperature. In general, under physiological conditions, we predict that fully-PS DNA oligonucleotides with T_m_ values within 0.25°C of 37°C can range in length from 13 to 26 nucleotides. Into the future, hybridization models rapidly determined by HRM involving RNA or other commonly used (and currently unmodeled modifications)–such as 2’-O-methoxyethyl, morpholino, and peptide nucleic acids–may also impact the efficient design of oligonucleotide therapeutics.

## Supporting information

S1 File Supporting information. Supporting text, tables, and figures as mentioned in the main text. This file includes sections describing the details of analyses performed and sequences used for the study, as well as additional analyses of data.
(PDF)

S2 File Supporting dataset 1. Melting temperature values as determined using high-resolution melting used to determine the hybridization models.
(XLSX)

S3 File Supporting dataset 2. Fitted thermodynamic values to the melting temperature data collected as shown in S2 File.
(XLSX)
