# Supplementary material for: Developing predictive hybridization models for phosphorothioate oligonucleotides using high-resolution melting
Source: PLoS One. 2022 May 18;17(5):e0268575. doi: 10.1371/journal.pone.0268575 (PMC9116672; doi:10.1371/journal.pone.0268575)
Supplement: S1 File — Supporting text, tables, and figures as mentioned in the main text. This file includes sections describing the details of analyses performed and sequences used for the study, as well as additional analyses of data. (PDF) [file pone.0268575.s001.pdf]

Supplementary Information

Developing predictive hybridization models for phosphorothioate oligonucleotides using high-resolution melting

Siyuan S. Wang[1,¶], Erhu Xiong[1,2,¶], Sanchita Bhadra[1], and Andrew D. Ellington[1,*]

[1] Department of Molecular Biosciences, Center for Systems and Synthetic Biology, College of Natural Sciences, The University of Texas at Austin, Austin, Texas, United State
[2] Current Address: School of Life Sciences, South China Normal University, Guangzhou, P.R. China

* Corresponding author
Email: ellingtonlab@gmail.com
¶ These authors contributed equally to this work.

Supplementary Text Section 1	Page 2	Description of nearest-neighbor models considered
Supplementary Text Section 2	Page 4	Leave-one-out cross validation analysis and error calculation
Table S1	Page 5	Sequences used for nearest-neighbor parameter determination
Table S2	Page 6	Partially and fully phosphorothioated sequences used to study partially-modified PS duplex stability
Table S3	Page 7	Sequences and domains used for high-temperature CHA
Table S4	Page 8	Sequences and domains used for low-temperature CHA
Table S5	Page 9	Non-salt-adjusted approximate parameters derived for PO-PO
Table S6	Page 10	Literature source and sequence of duplex stabilities as measured by UV-Vis
Table S7	Page 11	Non-salt-adjusted approximate parameters derived for PS-PS
Table S8	Page 12	Non-salt-adjusted approximate parameters derived for PS-PO
Figure S1	Page 13	Nearest-neighbor models considered and included variables
Figure S2	Page 14	Leave-one-out cross validation for PO-PO models
Figure S3	Page 15	Difference in T_m determined by HRM and by hyperchromicity models for all sequences
Figure S4	Page 16	Leave-one-out cross validation for PS-PS models
Figure S5	Page 17	Leave-one-out cross validation for PS-PO models

Section 1. Description of nearest-neighbor models considered

We considered several nearest-neighbor models for our analysis, which are described as follows. A visual of the parameters included for each model is shown in Supplementary Figure 1a. The following definitions and assumptions will be used:

- nearest-neighbor (NN) parameter - a variable representing the thermodynamic contribution of an internal nucleotide pair to the overall transition free energy (ΔG), enthalpy (ΔH), or entropy (ΔS) of the entire duplex
- nucleotide pair - two adjacent nucleotides with its complement that is considered as a thermodynamic parameter depending on the model
- terminal end - a single nucleotide paired to its complement at the 5' or 3' end of the duplex, represented with an adjacent "E" to denote the end of the strand
- For PS-PO duplexes, for all NN parameters there is a PS modification between the nucleotides in the top strand while the bottom nucleotide pair is unmodified

As an example, consider the PO-PO duplex $\begin{array}{l}\texttt{5' ATTTCGC 3'}\\ \texttt{\ \ \ |||||||}\\ \texttt{3' TAAAGCG 5'}\end{array}$ and its PS-PO counterpart $\begin{array}{l}\texttt{5' A*T*T*T*C*G*C 3'}\\ \texttt{\ \ \ | | | | | | |}\\ \texttt{3' T A A A G C G 5'}\end{array}$.

1. The asymmetrical model includes 16 NN parameters. 6 of these nucleotide pairs are non-unique; in other words, they have the same sequence as other pairs when the top and bottom strand are inverted (e.g. $\begin{array}{l}\texttt{5'AC}\\ \texttt{3'TG}\end{array}$ and $\begin{array}{l}\texttt{5'GT}\\ \texttt{3'AC}\end{array}$), but are treated as different variables under this model. The duplexes above can be represented as

$$\begin{array}{l}\texttt{5'AT}\\ \texttt{3'TA}\end{array} + \begin{array}{l}\texttt{5'TT}\\ \texttt{3'AA}\end{array} + \begin{array}{l}\texttt{5'TT}\\ \texttt{3'AA}\end{array} + \begin{array}{l}\texttt{5'TC}\\ \texttt{3'AG}\end{array} + \begin{array}{l}\texttt{5'CG}\\ \texttt{3'GC}\end{array} + \begin{array}{l}\texttt{5'GC}\\ \texttt{3'CG}\end{array} \quad \text{(for PO-PO)}$$

$$\begin{array}{l}\texttt{5'A*T}\\ \texttt{3'T A}\end{array} + \begin{array}{l}\texttt{5'T*T}\\ \texttt{3'A A}\end{array} + \begin{array}{l}\texttt{5'T*T}\\ \texttt{3'A A}\end{array} + \begin{array}{l}\texttt{5'T*C}\\ \texttt{3'A G}\end{array} + \begin{array}{l}\texttt{5'C*G}\\ \texttt{3'G C}\end{array} + \begin{array}{l}\texttt{5'G*C}\\ \texttt{3'C G}\end{array} \quad \text{(for PS-PO)}$$

and as a vector

$$\left[x_{\substack{AA\\TT}}, x_{\substack{AT\\TA}}, x_{\substack{AC\\TG}}, x_{\substack{AG\\TC}}, x_{\substack{TA\\AT}}, x_{\substack{TT\\AA}}, x_{\substack{TC\\AG}}, x_{\substack{TG\\AC}}, x_{\substack{CA\\GT}}, x_{\substack{CT\\GA}}, x_{\substack{CC\\GG}}, x_{\substack{CG\\GC}}, x_{\substack{GA\\CT}}, x_{\substack{GT\\CA}}, x_{\substack{GC\\CG}}, x_{\substack{GG\\CC}}\right]$$
$$= [0, 1, 0, 0, 0, 2, 1, 0, 0, 0, 0, 1, 0, 0, 1, 0]$$

This vector representation is the same for PO-PO, PS-PO, and PS-PS duplexes of the same sequence, while the parameter set changes (i.e. no PS modifications for PO-PO, 1 modification on the top strand for PS-PO, and 1 modification each for top and bottom strands for PS-PS). The asymmetrical model allows the duplex to have different chemical properties (e.g. of RNA-DNA, PS-PO); hence, that a nucleotide pair (e.g. $\begin{array}{l}\texttt{5'CT}\\ \texttt{3'GA}\end{array}$) can have a different impact on duplex stability than its inverse (e.g. $\begin{array}{l}\texttt{5'AG}\\ \texttt{3'TC}\end{array}$).

2. The symmetrical model includes 10 NN parameters that are a subset of the 16 in the asymmetrical model. This gives

$$\genfrac{}{}{0pt}{}{5'AT}{3'TA} + \genfrac{}{}{0pt}{}{5'AA}{3'TT}\left(\genfrac{}{}{0pt}{}{5'TT}{3'AA}\text{ flipped}\right) + \genfrac{}{}{0pt}{}{5'AA}{3'TT}\left(\genfrac{}{}{0pt}{}{5'TT}{3'AA}\text{ flipped}\right) + \genfrac{}{}{0pt}{}{5'GA}{3'CT}\left(\genfrac{}{}{0pt}{}{5'TC}{3'AG}\text{ flipped}\right) + \genfrac{}{}{0pt}{}{5'CG}{3'GC} + \genfrac{}{}{0pt}{}{5'GC}{3'CG} \text{ (for PO-PO)}$$

and as a vector

$$[x_{\substack{AA\\TT}}, x_{\substack{AT\\TA}}, x_{\substack{TA\\AT}}, x_{\substack{CA\\GT}}, x_{\substack{GT\\CA}}, x_{\substack{CT\\GA}}, x_{\substack{GA\\CT}}, x_{\substack{CG\\GC}}, x_{\substack{GC\\CG}}, x_{\substack{GG\\CC}}] = [2, 1, 0, 0, 0, 0, 1, 1, 1, 0]$$

Because this model assumes that the top and bottom nucleotide pairs have the same chemical properties, it should only be applied to chemically "symmetrical" duplexes (e.g. DNA-DNA, RNA-RNA, PS-PS).

3. The asymmetrical + terminal model includes the same 16 parameters as the asymmetrical model described above, plus all 8 possible terminal parameters:

$$\genfrac{}{}{0pt}{}{5'E\ A}{3'E'T} + \genfrac{}{}{0pt}{}{5'AT}{3'TA} + \genfrac{}{}{0pt}{}{5'TT}{3'AA} + \genfrac{}{}{0pt}{}{5'TT}{3'AA} + \genfrac{}{}{0pt}{}{5'TC}{3'AG} + \genfrac{}{}{0pt}{}{5'CG}{3'GC} + \genfrac{}{}{0pt}{}{5'GC}{3'CG} + \genfrac{}{}{0pt}{}{5'GE'}{3'CE} \text{ (for PO-PO)}$$

$$\genfrac{}{}{0pt}{}{5'E\ A}{3'E'T} + \genfrac{}{}{0pt}{}{5'A{*}T}{3'T\ A} + \genfrac{}{}{0pt}{}{5'T{*}T}{3'A\ A} + \genfrac{}{}{0pt}{}{5'T{*}T}{3'A\ A} + \genfrac{}{}{0pt}{}{5'T{*}C}{3'A\ G} + \genfrac{}{}{0pt}{}{5'C{*}G}{3'G\ C} + \genfrac{}{}{0pt}{}{5'G{*}C}{3'C\ G} + \genfrac{}{}{0pt}{}{5'GE'}{3'CE} \text{ (for PS-PO)}$$

and as a vector

$$[x_{\substack{AA\\TT}}, x_{\substack{AT\\TA}}, x_{\substack{AC\\TG}}, x_{\substack{AG\\TC}}, x_{\substack{TA\\AT}}, x_{\substack{TT\\AA}}, x_{\substack{TC\\AG}}, x_{\substack{TG\\AC}}, x_{\substack{CA\\GT}}, x_{\substack{CT\\GA}}, x_{\substack{CC\\GG}}, x_{\substack{CG\\GC}}, x_{\substack{GA\\CT}}, x_{\substack{GT\\CA}}, x_{\substack{GC\\CG}}, x_{\substack{GG\\CC}}, x_{\substack{EA\\ET}}, x_{\substack{AE\\TE}}, x_{\substack{ET\\EA}}, x_{\substack{TE\\AE}}, x_{\substack{EC\\EG}}, x_{\substack{CE\\GE}}, x_{\substack{EG\\EC}}, x_{\substack{GE\\CE}}]$$
$$= [0, 1, 0, 0, 0, 2, 1, 0, 0, 0, 0, 1, 0, 0, 1, 0, 1, 0, 0, 0, 0, 0, 0, 1]$$

4. The symmetrical + terminal model includes the same 10 rotationally unique internal parameters as the symmetrical model described above, plus an additional 4 terminal parameters selected out of the 8 possible terminal parameters such that no two parameters are inverses of one another (i.e. all parameters are unique). For example, either $\genfrac{}{}{0pt}{}{5'CE'}{3'GE}$ or $\genfrac{}{}{0pt}{}{5'E\ G}{3'E'C}$ could be included as a parameter, but not both as they are inverses of one another:

$$\genfrac{}{}{0pt}{}{5'E\ A}{3'E'T} + \genfrac{}{}{0pt}{}{5'AT}{3'TA} + \genfrac{}{}{0pt}{}{5'AA}{3'TT} + \genfrac{}{}{0pt}{}{5'AA}{3'TT} + \genfrac{}{}{0pt}{}{5'GA}{3'CT} + \genfrac{}{}{0pt}{}{5'CG}{3'GC} + \genfrac{}{}{0pt}{}{5'GC}{3'CG} + \genfrac{}{}{0pt}{}{5'CE'}{3'GE} \text{ (for PO-PO)}$$

and as a vector

$$[x_{\substack{AA\\TT}}, x_{\substack{AT\\TA}}, x_{\substack{TA\\AT}}, x_{\substack{CA\\GT}}, x_{\substack{GT\\CA}}, x_{\substack{CT\\GA}}, x_{\substack{GA\\CT}}, x_{\substack{CG\\GC}}, x_{\substack{GC\\CG}}, x_{\substack{GG\\CC}}, x_{\substack{EA\\ET}}, x_{\substack{AE\\TE}}, x_{\substack{EC\\EG}}, x_{\substack{CE\\GE}}]$$
$$= [2, 1, 0, 0, 0, 0, 1, 1, 1, 0, 1, 0, 0, 1]$$

Note that nucleotides at the termini (A at the 5' and C at the 3') are counted both as NN parameters ($\genfrac{}{}{0pt}{}{5'AT}{3'TA}$ and $\genfrac{}{}{0pt}{}{5'GC}{3'CG}$, respectively) and as terminal parameters ($\genfrac{}{}{0pt}{}{5'E\ A}{3'E'T}$ and $\genfrac{}{}{0pt}{}{5'CE'}{3'GE}$, respectively).

All models except the asymmetrical + terminal model can be fully determined since the maximum rank of the matrix of the sequence set is 20, which is larger than the number of variables included in those models.

Section 2. Leave-one-out cross validation analysis and error calculation

We performed leave-one-out-cross validation (LOOCV) on each dataset (i.e. PS-PS, PS-PO, PO-PO) for each of the 3 NN models. For each sequence, we trained the model on the remaining n-1 sequences and used the resulting model parameters to predict the ΔG. To compare the quality of prediction of each model, we calculated the root mean square error (RMSE) for both in-sample data (n-1 training sequences) and out-sample data (excluded sequence).

For each sequence i, let the observed ΔG be x_i, and the predicted ΔG be $\hat{x}_i$. Let the total number of predicted sequences be n. The RMSE is calculated as:

$$\text{RMSE} = \sqrt{\frac{\sum_i^n \left(x_i - \hat{x}_i\right)^2}{n}}$$

RMSE has the useful property that reported prediction errors are in the same units as that of the predicted quantity.

Number	Sequence	Number	Sequence
1	CTGTAAGGCGATATGTT	34	AATGTCGAACAGCAATT
2	TGCCATGTTGAAAACC	35	GTGAAACAATGCTGTAG
3	TATTCTGCCAATGGAAC	36	CAAGCCTCGATTTTGT
4	GGTTGCGGTGGCCAAC	37	GTGAGCAGAAGGGGTT
5	CGACATGTATGGCACAG	38	ACTCGCTCTACCTTAAT
6	CTCGGAGGCCCCATTTA	39	CTTTTGTGCGGGTAGC
7	CCGCTCAGAGTAGAGA	40	TTCGCGGTCTCCATTA
8	AATGAGGAGTGAAATGG	41	CGTGCAGCACTACTTG
9	GCTGAACTAACCACCA	42	GTCATTGTGCTTTTGC
10	CCACTAGCGGCGCCGTT	43	GAACCGTTGATGATCTC
11	GAGGCAGCCGCGACCTG	44	TGTCGCACCCTACTA
12	AGAGCGCCCTGCTGCC	45	TACTTCCAACGTAGG
13	GATGCCGCAGCGACCTG	46	ACGGGTCGTTCCGTG
14	AACGAATGTCAGCAATT	47	GTGGTACAAATGCGACC
15	GCTGTGAAACAATGTAG	48	AGCACGGTGGTACAACA
16	CGATTTTGTCAAGCCT	49	GGTGGCGTTCTT
17	GAGCAGAAGGGGTTGT	50	TCTACACCGCGA
18	ATAACTTACTCTCGCCT	51	ACTGTATCGCCCTA
19	CGGTGCTTTTGGTAGC	52	CCGTTGCTGCTAGG
20	TCTCGCGGTTCCATTA	53	TAGACGCGGCCTCTTTCC
21	CGTGTGGATAATTAGCT	54	CTAAACTGTTATAGCCGG
22	TTGAAAACCCATGTGC	55	TGTAAGACTTCTGCCAGAAA
23	TTAACCTGCAATGGATC	56	CGCGCGAGTATTTATAACCT
24	GCGGTTGGTGGCCAAC	57	TTCTACATCCATCTTAATCCCA
25	CACAGCGACATGTATGG	58	AGACATCCCATACGAGCATCCA
26	CCCAGGCCTCGGATTTA	59	ACATGACTCATCTTAGCCGGCGAG
27	CTCAGAGTAGAGCCGA	60	CGGGATTTCTGGCATCATTGTCCT
28	AGGAGTGAATGAAATGG	61	TAATTATACGAGTAGTTTCTGTCCTG
29	GAACTGCCACTACCAA	62	GATTGTATCATCGACATCACACTACC
30	CTACCAGCGGCGCCGTT	63	CAAACTTAGTAATCACGCCCAGCAACCA
31	GCAGGAGCCGCGACCTG	64	GATCTCTCTATCATCGTTTATTGGGTAT
32	AGCTGAGCCTGCCGCC	65	TTGTAGTTGACGTTTGTGATTTAGTGAATT
33	GCCCGAGCATGACTGCG	66	TTTGGGTTAGTAAGAAGGCAGCAGTTGGGC

Table S1. Sequences used for model parameter determination. Sequences were hybridized to their complements prior to HRM. Fully phosphodiester and fully phosphorothioate versions of each sequence listed were used in the study. Sequences maximally span the space of nearest neighbor pairs (i.e. sequences are maximally independent).

Name	Sequence
Seq1	GCCATCTACTCATACCTAAC
1PS-Seq1	G*CCATCTACTCATACCTAAC
4PS-Seq1	G*C*C*A*TCTACTCATACCTAAC
9PS-Seq1	G*C*C*A*T*C*T*A*C*TCATACCTAAC
PS-Seq1	G*C*C*A*T*C*T*A*C*T*C*A*T*A*C*C*T*A*A*
Seq1Comp	GTTAGGTATGAGTAGATGGC
PS-Seq1Comp	G*T*T*A*G*G*T*A*T*G*A*G*T*A*G*A*T*G*G*
Seq2	GCAAGTTCAGGTCAGGTATC
1PS-Seq2	G*CAAGTTCAGGTCAGGTATC
4PS-Seq2	G*C*A*A*GTTCAGGTCAGGTATC
9PS-Seq2	G*C*A*A*G*T*T*C*A*GGTCAGGTATC
PS-Seq2	G*C*A*A*G*T*T*C*A*G*G*T*C*A*G*G*T*A*T*
Seq2Comp	GATACCTGACCTGAACTTGC
PS-Seq2Comp	G*A*T*A*C*C*T*G*A*C*C*T*G*A*A*C*T*T*G*

Table S2. Partially and fully phosphorothioate-modified sequences used to study partially-modified PS duplex stability. Comp = complement. Asterisks in sequence denote positions in the sequence backbone with PS modifications.

HT-CHA	Sequence
HT-H1	GTCACGTGA GCTAGCGTT AGCATCGTCG CCATGCTGCTAGCA CGACGATGCT AACGCTAGC CCTTGTCA TACGCAGCAC
HT-H1-PSall	G*T*C*A*C*G*T*G*A* G*C*T*A*G*C*G*T*T* A*G*C*A*T*C*G*T*C*G* C*C*A*T*G*C*T*G*C*T*A*G*C*A* C*G*A*C*G*A*T*G*C*T* A*A*C*G*C*T*A*G*C* C*C*T*T*G*T*C*A* T*A*C*G*C*A*G*C*A*C
HT-H2	AGCATCGTCG TGCTAGCAGCATGG CGACGATGCT AACGCTAGC CCATGCTGCTAGCA
HT-H2-PSall	A*G*C*A*T*C*G*T*C*G* T*G*C*T*A*G*C*A*G*C*A*T*G*G* C*G*A*C*G*A*T*G*C*T* A*A*C*G*C*T*A*G*C* C*C*A*T*G*C*T*G*C*T*A*G*C*A
HT-Catalyst	CGACGATGCT AACGCTAGC TCACGTGAC
HT-RF	/56-FAM/CGA GTGCTGCGTA TGACAAGG GCTAGCGTT
HT-RQ	C CCTTGTCA TACGCAGCAC TCG /3IABkFQ/
HT-Domain 1	TCACGTGAC
HT-Domain 2	AACGCTAGC
HT-Domain 3	CGACGATGCT
HT-Domain 4	CCATGCTGCTAGCA
HT-Domain 5	CCTTGTCA
HT-Domain 6	TACGCAGCAC

Table S3. Sequences and domains used for high-temperature CHA. Asterisks in sequence indicate positions with PS backbones. Different domains are indicated by different colors. /56-FAM/ = 5’ Fluorescein; /3IABkFQ/ = 3’ Iowa Black FQ.

LT-CHA	Sequence
LT-H1	GTCAGTGA GCTAGGTT AGATGTCG CCATGTGTAGA CGACATCT AACCTAGC CCTTGTCA TAGAGCAC
LT-H1-PS1	G*T*C*A*G*T*G*A GCTAGGTT AGATGTCG CCATGTGTAGA CGACATCT AACCTAGC CCTTGTCA TAGAGCAC
LT-H2	AGATGTCG TCTACACATGG CGACATCT AACCTAGC CCATGTGTAGA
LT-H2-PS3	A*G*A*T*G*T*C*G TCTACACATGG CGACATCT AACCTAGC CCATGTGTAGA
LT-Catalyst	CGACATCT AACCTAGC TCACTGAC
LT-Catalyst-PS1	CGACATCT AACCTAGC T*C*A*C*T*G*A*C
LT-Catalyst-PS2	CGACATCT A*A*C*C*T*A*G*C* TCACTGAC
LT-Catalyst-PSall	C*G*A*C*A*T*C*T* A*A*C*C*T*A*G*C* T*C*A*C*T*G*A*C
LT-RF	/56-FAM/CGA GTGCTCTA TGACAAGG GCTAGGTT
LT-RQ	C CCTTGTCA TAGAGCAC TCG /3IABkFQ/
LT-Domain 1	TCACTGAC
LT-Domain 2	AACCTAGC
LT-Domain 3	CGACATCT
LT-Domain 4	CCATGTGTAGA
LT-Domain 5	CCTTGTCA
LT-Domain 6	TAGAGCAC

Table S4. Sequences and domains used for low-temperature CHA. Asterisks in sequence indicate positions with PS backbones. Different domains are indicated by different colors. /56-FAM/ = 5' Fluorescein; /3IABkFQ/ = 3' Iowa Black FQ.

	Non-salt-adjusted PO-PO parameters		
**Nucleotide Pairs**	**ΔG_{50} (kcal/mol)**	**ΔH (kcal/mol)**	**ΔS (cal/K/mol)**
AA/TT	-0.74±0.14	-8.10±1.68	-22.8±4.8
AT/TA	-0.47±0.10	-5.53±1.35	-15.6±3.9
TA/AT	-0.50±0.12	-6.40±1.49	-18.3±4.3
CA/GT	-0.87±0.15	-6.89±1.70	-18.6±4.8
GT/CA	-0.85±0.15	-7.12±1.88	-19.4±5.3
CT/GA	-0.85±0.14	-7.51±1.63	-20.6±4.6
GA/CT	-0.80±0.14	-6.51±1.84	-17.7±5.3
CG/GC	-1.53±0.16	-10.81±2.03	-28.7±5.8
GC/CG	-1.67±0.16	-12.68±1.98	-34.1±5.6
GG/CC	-1.00±0.15	-6.09±1.71	-15.8±4.8
EA/ET	0.54±0.40	20.73±4.98	62.5±14.2
AE/TE	0.52±0.40	20.20±4.89	60.9±13.9
EC/EG	0.67±0.40	21.07±4.93	63.1±14.0
CE/GE	0.47±0.40	18.09±4.84	54.5±13.8

Table S5. Non-salt-adjusted approximate parameters derived from HRM data for native DNA duplexes. All reported values are adjusted to 1 M NaCl and 50°C. PO-PO = Phosphodiester-phosphodiester duplexes. Errors are defined as the standard deviations of the parameter fits. Parameter values are non-unique solutions from the model fit.

#	Source	Sequence	ΔG_{37} (kcal/mol)
1	SantaLucia Biochemistry 1996	CCATCGCTACC	-13.3
2	Sugimoto NAR 1996	CGGCAAGCGC	-13.3
3	Breslauer PNAS 1986	GCGAATTCGC	-12.9
4	SantaLucia Biochemistry 1996	CATATGGCCATATG	-12.7
5	SantaLucia Biochemistry 1996	CTGACAAGTGTC	-12.6
6	SantaLucia Biochemistry 1996	CAACTTGATATTATTA	-12.4
7	SantaLucia Biochemistry 1996	CCATTGCTACC	-12.2
8	Owczarzy Biopolymers 1997*	GACGTGTGAC	-12.1
9	Sugimoto NAR 1996	ACATTATTATTACA	-11.3
10	Owczarzy Biopolymers 1997*	ACAGTGACAC	-11.3
11	Sugimoto NAR 1996	ACGTATTATGC	-10.4
12	Sugimoto NAR 1996	ATTGGATACAAA	-10.3
13	SantaLucia Biochemistry 1996	ATGAGCTCAT	-10.2
14	Owczarzy Biopolymers 1997*	GTAGTAGTAG	-8.46
15	Sugimoto NAR 1996	TAGGTTATAA	-7.0
16	Owczarzy Biopolymers 1997*	CATATATATG	-5.74

Table S6. Literature sources and sequences of duplex stabilities as measured by UV-Vis. All melting experiments were performed in 1 M sodium; predictions from HRM-derived parameters were adjusted to 1 M sodium as well. Sources reported ΔG extrapolated to 37°C for all sequences, with the exception of sources marked with an asterisk, for which ΔH and ΔS values were used to calculate ΔG_{37}.

	Non-salt-adjusted PS-PS parameters		
**Nucleotide Pairs**	**ΔG_{50} (kcal/mol)**	**ΔH (kcal/mol)**	**ΔS (cal/K/mol)**
AA/TT	-0.18±0.03	-4.36±0.77	-13.0±2.3
AT/TA	-0.08±0.02	-3.64±0.52	-11.0±1.5
TA/AT	-0.03±0.01	-1.93±0.53	-5.9±1.6
CA/GT	-0.44±0.02	-5.52±0.57	-15.7±1.7
GT/CA	-0.41±0.03	-3.95±0.75	-11.0±2.3
CT/GA	-0.42±0.03	-4.16±0.64	-11.6±1.9
GA/CT	-0.48±0.03	-5.07±0.90	-14.2±2.7
CG/GC	-0.97±0.04	-6.16±1.01	-16.1±3.0
GC/CG	-0.95±0.04	-6.90±0.77	-18.4±2.3
GG/CC	-0.77±0.03	-5.09±0.83	-13.4±2.5
EA/ET	-1.26±0.08	3.24±2.05	13.9±6.2
AE/TE	-1.24±0.08	4.18±2.02	16.8±6.1
EC/EG	-1.16±0.08	1.47±1.98	8.1±5.9
CE/GE	-1.16±0.08	0.64±2.05	5.6±6.1

Table S7. Non-salt-adjusted approximate parameters derived from HRM data for phosphorothioate-phosphorothioate (PS-PS) DNA duplexes. All reported values are adjusted to 1 M NaCl and 50°C. Errors are defined as the standard deviations of the parameter fits. Parameter values are non-unique solutions from the model fit.

	Non-salt-adjusted PS-PO parameters		
**Nucleotide Pairs**	**ΔG_{50} (kcal/mol)**	**ΔH (kcal/mol)**	**ΔS (cal/K/mol)**
AA/TT	-0.44±0.20	-5.81±3.12	-16.6±9.1
AT/TA	-0.33±0.10	-5.64±1.69	-16.4±4.9
AC/TG	-0.80±0.11	-8.66±1.85	-24.3±5.4
AG/TC	-0.63±0.09	-6.13±0.85	-17.0±2.3
TA/AT	-0.22±0.10	-3.86±1.90	-11.3±5.6
TT/AA	-0.40±0.07	-5.87±0.64	-16.9±1.7
TC/AG	-0.56±0.16	-6.13±2.71	-17.2±7.9
TG/AC	-0.69±0.15	-7.28±2.75	-20.4±8.0
CA/GT	-0.74±0.17	-7.23±2.67	-20.1±7.7
CT/GA	-0.58±0.19	-6.30±3.21	-17.7±9.4
CC/GG	-0.82±0.11	-5.57±1.57	-14.7±4.5
CG/GC	-1.13±0.18	-8.07±3.09	-21.5±9.0
GA/CT	-0.77±0.12	-7.92±2.38	-22.1±7.0
GT/CA	-0.52±0.14	-5.46±2.27	-15.3±6.6
GC/CG	-1.07±0.15	-6.63±2.26	-17.2±6.6
GG/CC	-1.01±0.17	-7.75±2.81	-20.8±8.2
EA/ET	-0.53±0.40	13.64±6.63	43.8±19.3
AE/TE	-0.49±0.37	15.07±6.01	48.2±17.5
ET/EA	-0.54±0.39	14.87±6.29	47.7±18.3
TE/AE	-0.52±0.38	14.72±6.18	47.2±18.0
EC/EG	-0.53±0.33	10.31±5.35	33.5±15.6
CE/GE	-0.51±0.38	10.49±6.30	34.0±18.4
EG/EC	-0.28±0.40	16.13±6.63	50.8±19.3
GE/CE	-0.37±0.39	14.65±6.49	46.5±18.9

Table S8. Non-salt-adjusted approximate parameters derived from HRM data for phosphorothioate-phosphodiester (PS-PO) DNA duplexes. All reported values are adjusted to 1 M NaCl and 50°C. All internal nucleotide parameters have a PS linkage between the top nucleotide pair (e.g. 5'A*A/3'TT). Errors are defined as the standard deviations of the parameter fits. Parameter values are non-unique solutions from the model fit.

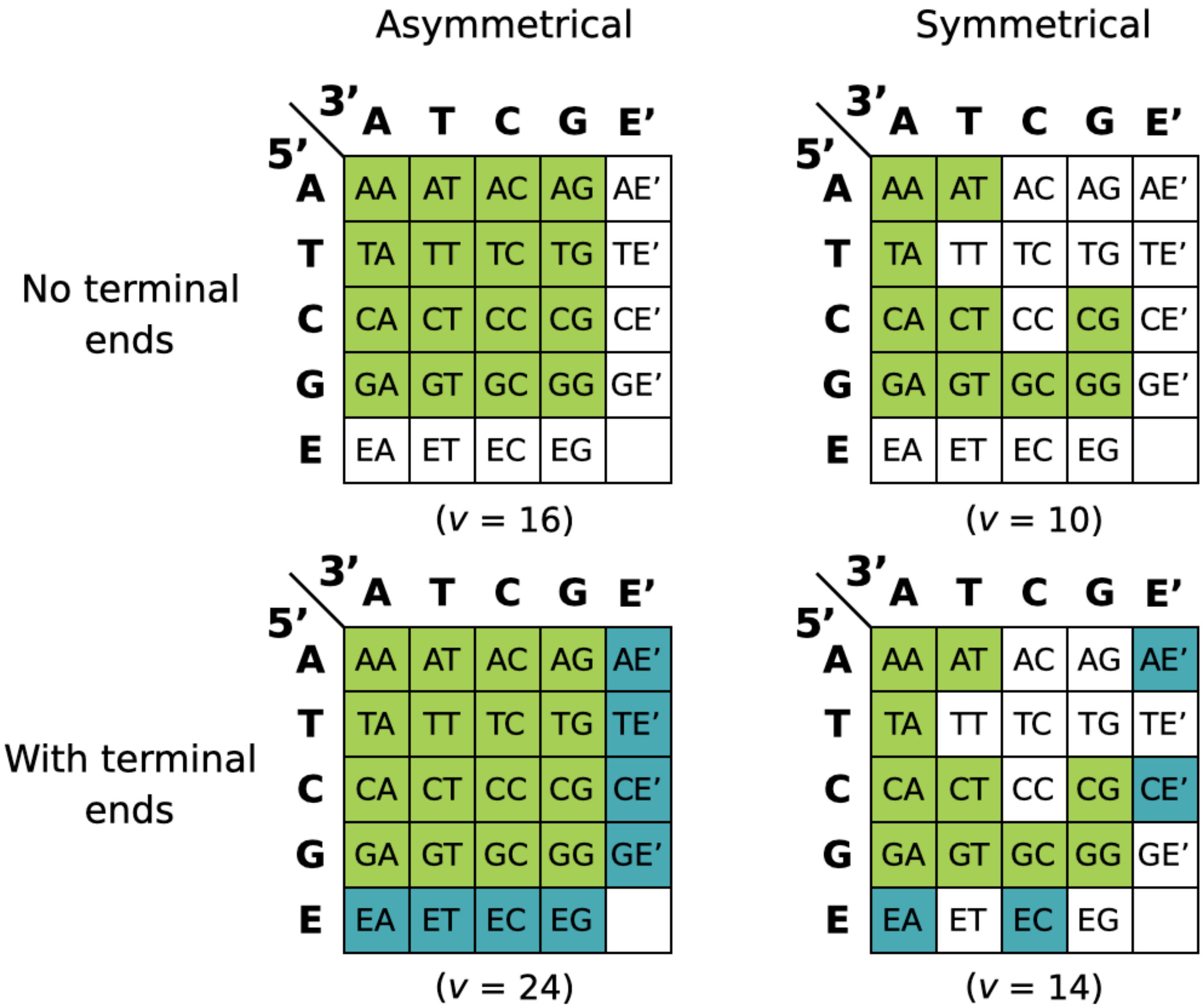

Figure S1. Nearest-neighbor style models considered and the parameters included in each model. Filled cells indicate that the nearest-neighbor pair was added as a variable to the model. Green = nucleotide pair variable. Blue = terminal nucleotide variable. v = total variables involved.

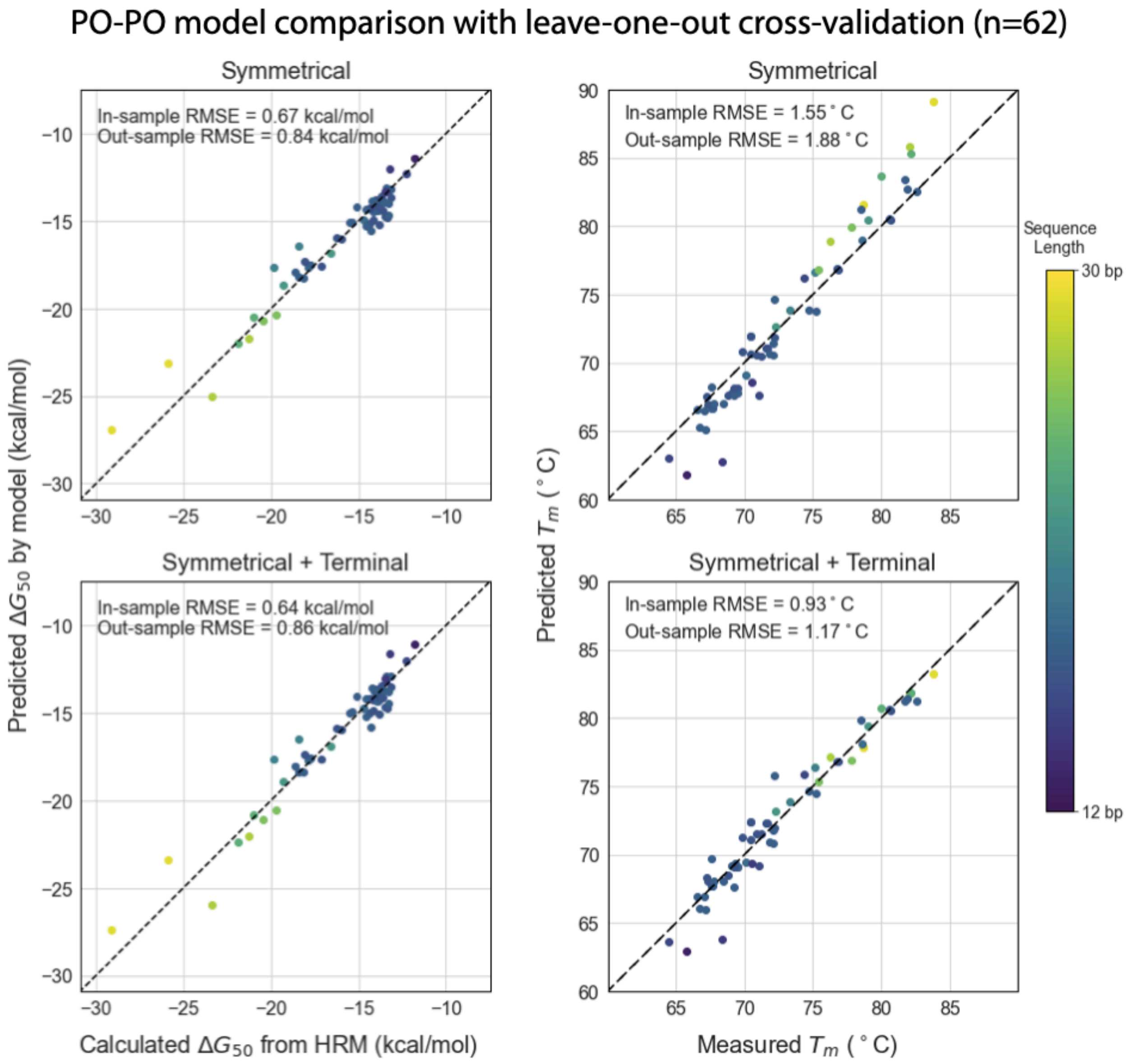

Figure S2. Leave-one-out cross-validation on the PO-PO HRM dataset for ΔG_{50} and T_m (concentration = 10 μM) without (top row) and with (bottom row) inclusion of terminal nucleotide variables. Color of dots represents the length of the sequence. The dashed line $y = x$ is added to guide the eye. RMSE = root mean square error.

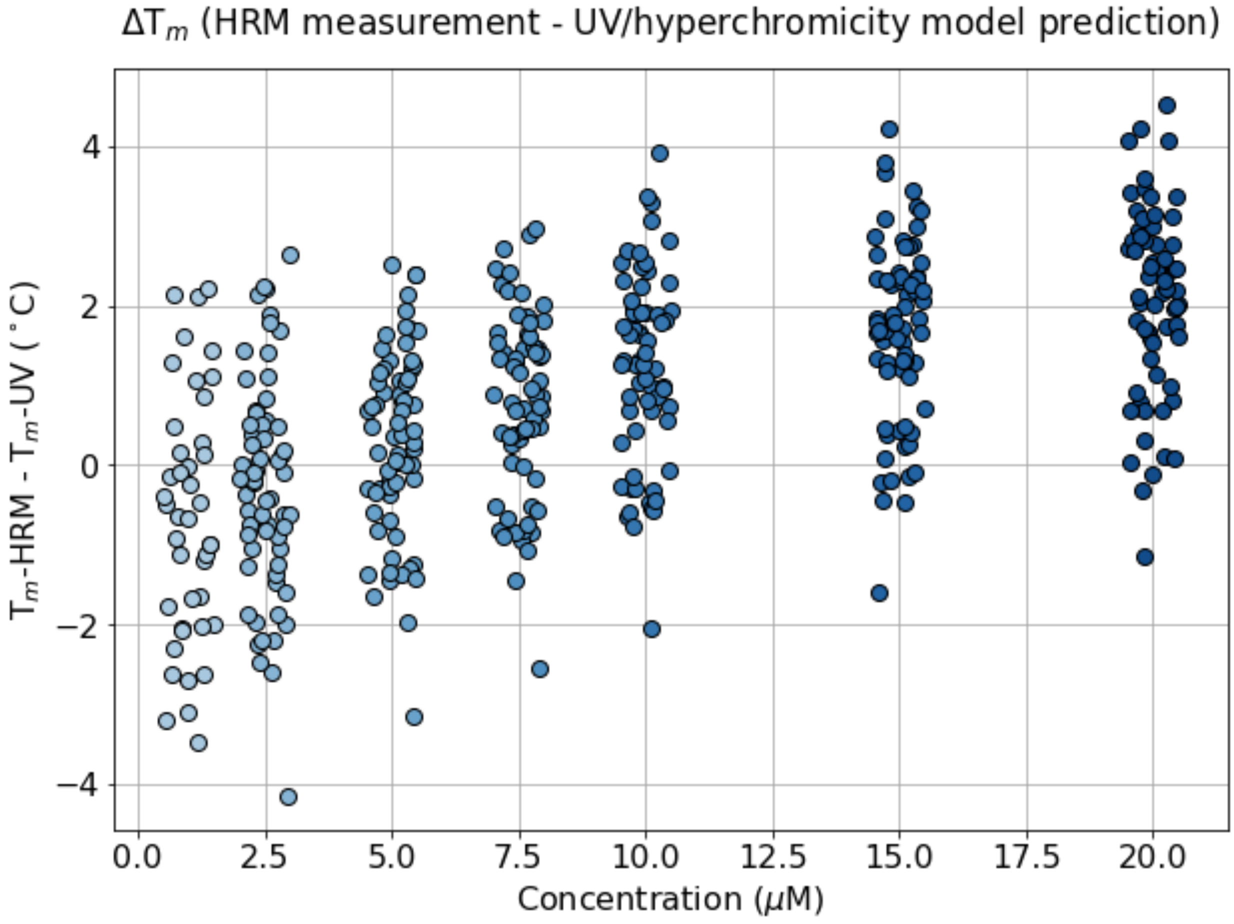

Figure S3. Difference in T_m determined by HRM and by hyperchromicity models for all sequences. Predictions were made using the model presented in [5].

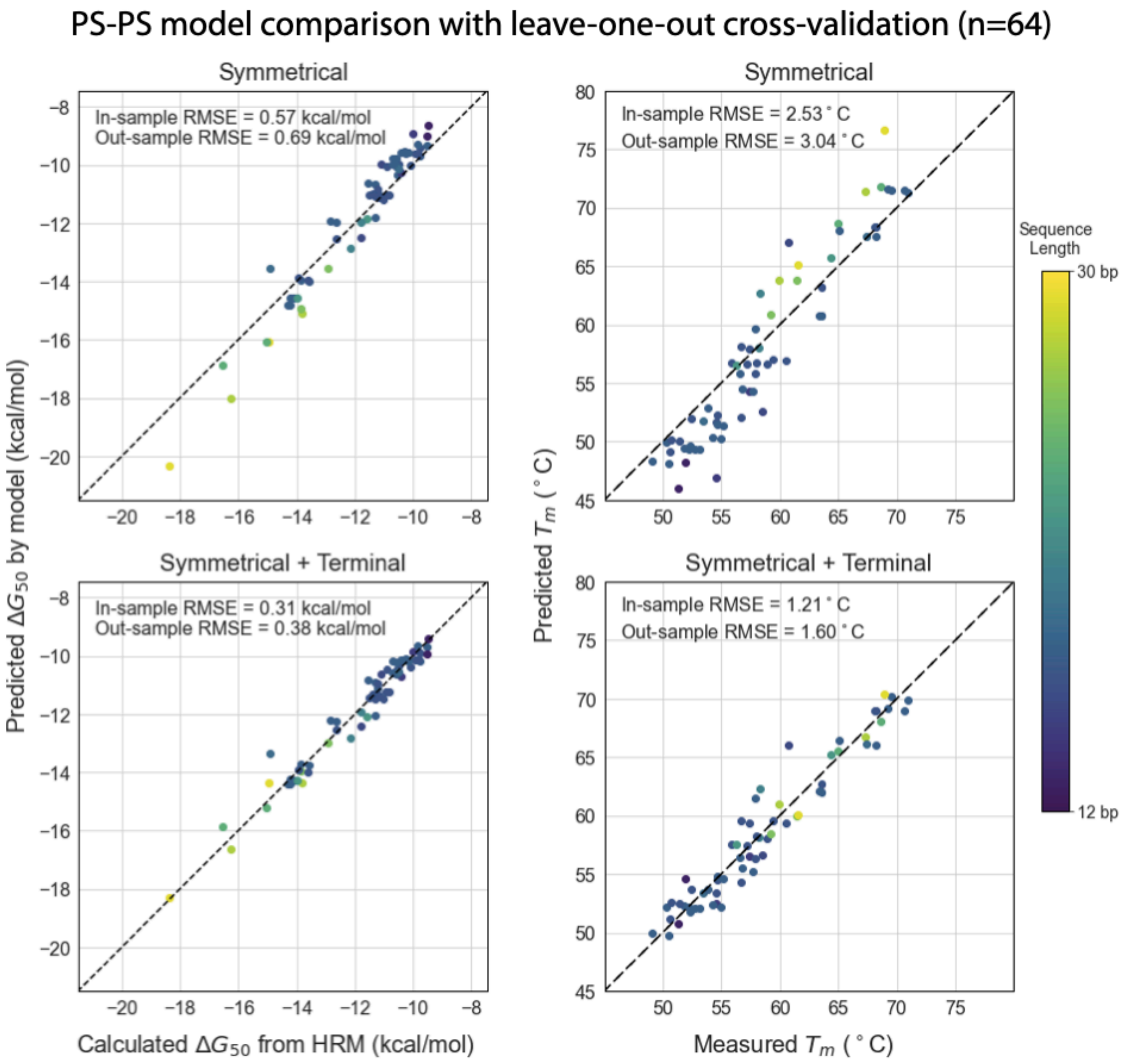

Figure S4. Leave-one-out cross-validation on the PS-PS HRM dataset for ΔG_{50} and T_m (concentration = 10 μM) without (top row) and with (bottom row) inclusion of terminal nucleotide variables. Color of dots represents the length of the sequence. The dashed line $y = x$ is added to guide the eye. RMSE = root mean square error.

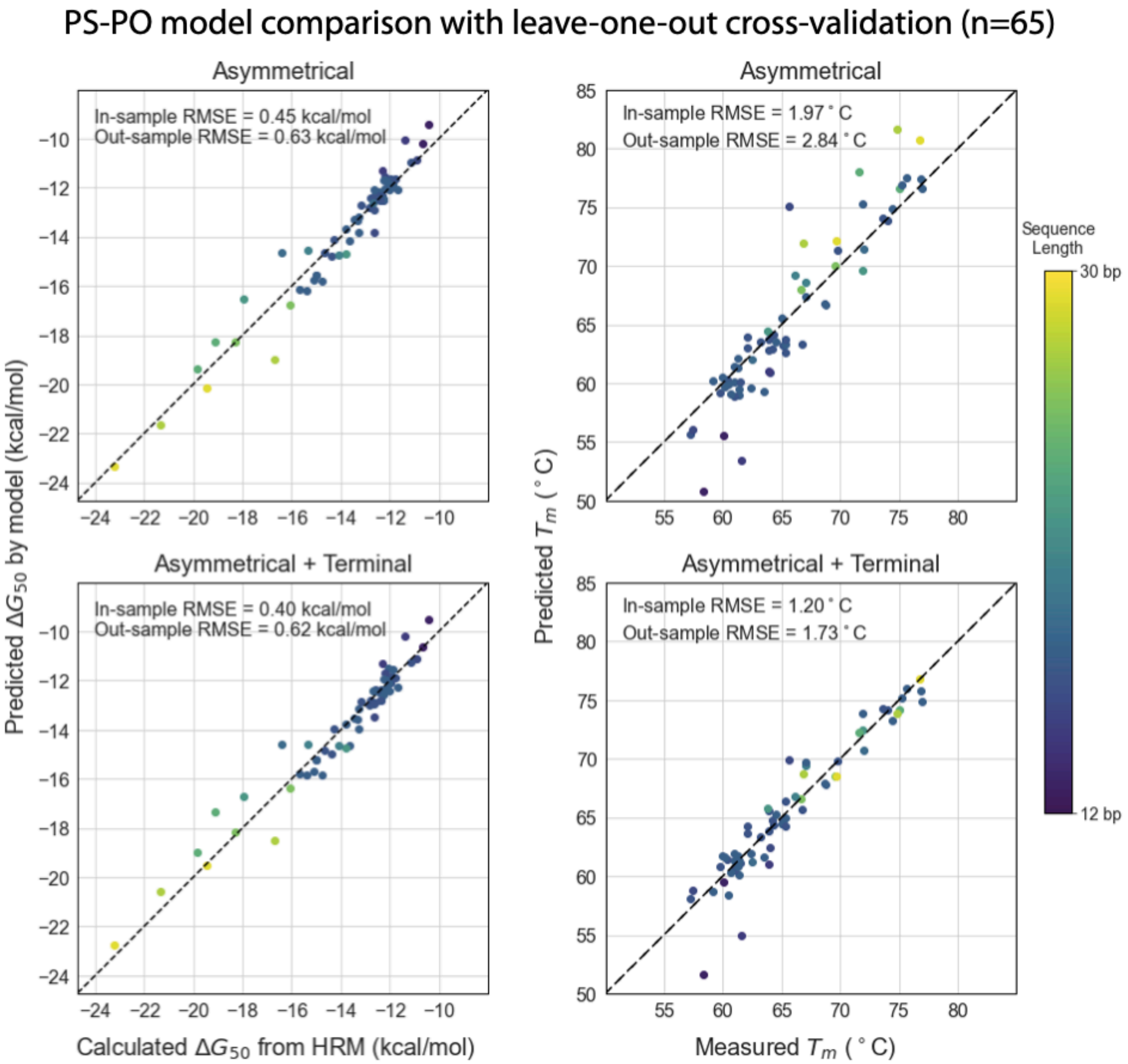

Figure S5. Leave-one-out cross-validation on the PS-PO HRM dataset for ΔG_{50} and T_m (concentration = 10 μM) without (top row) and with (bottom row) inclusion of terminal nucleotide variables. Color of dots represents the length of the sequence. The dashed line $y = x$ is added to guide the eye. RMSE = root mean square error.
